# Eclecticism vs. Homœopathy

**Published:** 1885-08

**Authors:** Ad. Lippe

**Affiliations:** Philadelphia


### ECLECTICISM *vs.* HOMŒOPATHY.

AD. LIPPE, M. D., PHILADELPHIA.

On page 196 of the *New England Medical Gazette*, a monthly journal of homœopathic medicine, May, 1885, we find a singular paper. It appears that Dr. R. R. Gregg's pamphlet on *Diphtheria* was translated into the French language, was read before *La Société Médicale Homœopathique de France*, by Dr. Charles Ozanam, the translator, and was published in its *Bulletin*. This happened in October, 1884. In November, 1884, Drs. Jousset, Piedvache, Cretin, and Claude, reported to be prominent as conservative and scientific thinkers, were indignant that this Society should offer to the profession in France, with the seal upon it of publication in the Society's official organ, a paper so pathologically weak and clinically absurd as Dr. Gregg's treatise. Spirited addresses were made, then and there, by Drs. Cretin and Jousset, and on motion of the former, the following resolution was adopted:

" *La Société Médicale Homœopathique de France*, looking upon the teaching of Drs. Gregg and A. Lippe as not only *bizarre* in itself, but as contrary to Homœopathy, regrets the publicity given in its *Bulletin* to such teaching and its applications."

And now comes this so-called expounder of homœopathic doctrine—the *New England Medical Gazette*—and indulges in the following comments:

" It is needless to say that it is the teaching of Dr. Gregg, and by no means Dr. Gregg himself, whose repudiation by our French colleagues we look upon as matter for sincere rejoicing. As we took occasion to point out in our issue for March last, the gulf between Homœopathists and Hahnemannians is widening rapidly year by year; and for every thoughtful and scientific man who frankly enrolls himself under the banner of the former, as opposed to the latter faction, Homœopathy has good reason to congratulate itself."

We can only express our sincere regret that *La Société Médicale Homœopathique*, in true French fashion, did change its mind so suddenly, first, in giving publicity through their acknowledged organ, the *Bulletin*, to Dr. Gregg's most excellent little work on *Diphtheria* and the true homœopathic treatment of this virulent disease in October, and a month later, induced by Drs. Cretin and Jousset, passing a resolution not only *bizarre*

but anti-homœopathic. When Dr. Cretin declares that the *ultra-infinitesimal* dilutions have not any medicinal action whatsoever he becomes *insulting*. Can Dr. Cretin suppose for a moment that his mere assertion will destroy the testimony of *hundreds* of thoughtful and observing men who have certified for many long years that the so-called high potencies are by far more efficacious than the lower dilutions for the cure of the sick? The ravings in which Dr. Cretin indulges are not arguments. The only argument possible to determine the posological question is "the results of experiments" by persons fully capable of making the experiments. Assertions are not arguments.

We now entertain the hope that Drs. Cretin and Jousset were proving Cannabis indica when they indulged in the luxury of dreaming aloud at that eventful November meeting of *La Société Médicale Homœopathique de France*, and that in the course of time they will cease dreaming.

*The New England Medical Gazette* informs us "that the gulf between Homœopathists and Hahnemannians is widening year by year." The learned editor of that journal seems to imply that the faction he represents are Homœopathists and not Hahnemannians. Hahnemann was the founder of a Healing Art he called Homœopathy, by its name proclaiming that the Law of the Similars was the only law of cure, and he only claims that he discovered the true method of applying this immutable law of nature for the cure of sick. He did not call his Healing Art "Hahnemannism," for the simple reason that the Law of the Similars was as an old law, as old as creation; that it was known to the earliest medical writers, but they did not know how to apply the law for the cure of the sick. The simple question is, can the Homœopathic Healing Art be successfully practiced without a compliance with the methods of Hahnemann? Certainly not. A Hahnemannian, according to the only definition of such a person, would be one who considers our Healing Art as a finished method of cure, finished by its founder. Such a person does not exist. As all arts are capable of further development, so must the Healing Art be capable of unceasing development. This capacity implies adherence to the methods of Hahnemann. A development is only possible by extending the methods of its founder, and we refer here to one of the last sentences the father of our school in this country, the late Dr. Hering, wrote: "*If our school ever gives up the strict inductive method of Hahnemann, we are lost, and deserve only to be mentioned as a caricature in the history of medicine.*" If the learned editor of *The New England Medical Gazette* considers and calls all homœopathists, who accept the strict inductive method of Hahnemann, Hahnemannians, he commits a fatal error, they are homœopathicians, healers who not only accept Hahnemann's methods, but constantly practice accordingly.

We fully understand the aims of that faction *The New England Medical Gazette* represents. We are also very much gratified to learn that the gulf between the homœopathists and non-homœopathists is widening year by year. That same faction existed in Hahnemann's days, and he called them "*pretenders*." The gulf between the homœopathicians and non-homœopathic eclectics has been widening, and continues to widen at the same ratio as these pretenders violate the methods of Hahnemann. That these men are eclectics to all intents and purposes is self-evident. We never deal in assertions; that is the perogative of the weak defenders of a bad and pernicious cause. We always offer substantial, *documentary evidence.*

On page 217 of the above-quoted journal we find these pretenders fully and irretrievably committed to eclecticism in its ugliest form.

The lecturer on Homœopathy there gives testimony which in no possible way can be construed into an acquittal of our accusations. He says: "As a matter of right, a homœopath should reserve unto himself the use of remedies according to other methods; for he always sees with satisfaction and encourages the employment of homœopathic remedies on the part of *regular* physicians." What, in the name of common sense, has become of the strict inductive method of Hahnemann? What has become of common logic? Because the "regular" physicians employ homœopathic remedies, we, as homœopaths, may have the right to use medicines according to other methods. The "regular" physician is one thing, and are *we*, as homœopaths, "irregular" physicians? We guess not; it is all the other way. That the physicians of the common school are learning to use our remedies in our preparations under the Law of the Similars is true—is a fact—and therefore retrogressive pretenders reserve unto themselves the right to pick up the remedies abandoned by the progressive allopathist. Absurd as this proposition is, the absurdity becomes still more glaring and offensive when these men claim the right to use remedies according to other methods. The methods of the common school of medicine and the methods of the Homœopathic Healing Art are absolutely antagonistic one to another; they have nothing in common, and how an intelligent physician can address himself to logical people as an advocate of a practice so mixed up must really appear as an incredible absurdity. If the Homœopathic Healing Art is true, how then can another method be also true? The aim of these illogical men who are incapable of mastering the Homœopathic Healing Art is to appear as "liberals," as true eclectics, who will resort to any method of cure which will momentarily satisfy the sick; and in order to improve their liberal eclectic position they lustily abuse both the consistent allopaths and homœopaths. They say: "But the occasional use of allopathic medicines has been met by the *regular school*, with the argument that homœopaths applying other than strictly homœopathic remedies are guilty of inconsistency and wrong-doing. Such objections belong to the same category as those of the dogmatic minority of homœopaths. It is here that extremes meet and display their absurdities." So the lecturer says. Absurdities, indeed—and the dogmatic minority are as guilty of absurdly rejecting pretending homœopaths on account of their palpable inconsistency as are the regulars. If an allopath uses homœopathic remedies, he impliedly admits that his own school has in that instance failed and he is progressive. If a pretender resorts to other methods than those of the school he professes to belong to, he impliedly admits that as far as he is personally concerned he does not know how to apply the methods of his school and flies to others in desperation. He deposits before the medical world his *testimonium paupertatis*, and he joins the out-and-out eclectics, but, with unparalleled effrontery, he claims to be a homœopath, and claims the freedom to do just as he pleases; defies the regulars and kicks against the homœopathicians as being in a dogmatic minority. Glad to hear from The Hub that the breach is widening, and it will keep on widening if such *absurd* lectures on Homœopathy are published in *professedly* homœopathic journals, as *The New England Gazette.* May the abettors and defenders of these absurd doctrines promulgated in this *Gazette* go on just as fast as they can. Let them be sure of a majority who will become full-fledged eclectics and follow the honest example of the New York journal, drop the name, as you have acknowledged your incapacity of rightly applying the homœopathic methods for curing the sick. Just here we would remind these eclectics of one fact they seem to have entirely overlooked and which they have omitted to show, viz.: the superior results of the eclectic mixed methods of cure over the results obtained by the dogmatic minority of homœopaths (as they insultingly style them). We have time and again implored these bold and abusive pretenders to publish but *one single* case of sickness in which they have treated the sick strictly homœopathically, and, failing to cure, have fallen back on other (eclectic) methods and then cured. These pretenders also seem ignorant of the fact that there are, sitting as judges, the most deeply interested party, *The People*. *They* observe the *results* of the treatment given them by the dogmatic homœopaths and by the pretenders. If the pretenders and eclectics are more successful than are the dogmatic homœopaths, the latter will have to subside, of course. For the present it looks very much the other way, and it does not matter how boastingly the eclectics blab about their rights to do just as they please, abuse those whom they thereby foolishly believe to be able to drive into their ranks. There are the judges, "the people." Their judgment is final. The case is before them; it is labeled "Eclecticism *vs.* Homœopathy."

